# Mycoplasma Contamination of Cell Cultures: Vesicular Traffic in Bacteria and Control over Infectious Agents

**Published:** 2014

**Authors:** V. M. Chernov, O. A. Chernova, J. T. Sanchez-Vega, A. I. Kolpakov, O. N. Ilinskaya

**Affiliations:** Kazan Institute of Biochemistry and Biophysics, Kazan Scientific Center, Russian Academy of Sciences, Lobachevskogo Str., 2/3, 1420111, Kazan, Russia; Kazan (Volga Region) Federal University, Kremlyovskaya Str., 18, 420008, Kazan, Russia; National Autonomous University of Mexico, Coyoacán, 04510, Mexico

**Keywords:** diagnosis and eradication, cell cultures, mycoplasma contamination

## Abstract

Cell cultures are subject to contamination either with cells of other cultures or with microorganisms, including fungi, viruses, and bacteria. Mycoplasma contamination of cell cultures is of particular importance. Since cell cultures are used for the production of vaccines and physiologically active compounds, designing a system for controlling contaminants becomes topical for fundamental science and biotechnological production. The discovery of extracellular membrane vesicles in mycoplasmas makes it necessary to take into consideration the bacterial vesicular traffic in systems designed for controlling infectious agents. The extracellular vesicles of bacteria mediate the traffic of proteins and genes, participate in cell-to-cell interactions, as well as in the pathogenesis and development of resistance to antibiotics. The present review discusses the features of mycoplasmas, their extracellular vesicles, and the interaction between contaminants and eukaryotic cells. Furthermore, it provides an analysis of the problems associated with modern methods of diagnosis and eradication of mycoplasma contamination from cell cultures and prospects for their solution.

## INTRODUCTION

With the use of cell cultures expanding in fundamental and practical studies, it is utterly important to elaborate a system for rigorous testing of any contamination of the material. Working with cell cultures always presents a risk of contamination either with eukaryotic cells from other cultures or with microorganisms, including fungi, viruses and bacteria. Mycoplasma contamination is of particular preoccupation as it does not manifest itself conspicuously [1-3].

In 1956, for the purpose of investigating the effects of mycoplasma on eukaryotic cells, Robinson *et al*. infected cell cultures with these microorganisms. They found that the original cell culture had already been contaminated with mycoplasma. This was the first report on the detection of mycoplasma in cell cultures [4]. Subsequently, it became clear that mycoplasma contamination is the scourge of cell cultures. It turns out that all cell cultures originating from various eukaryotic organisms (mammals, birds, reptiles, fishes, insects and plants) are subject to mycoplasma contamination. Experimental studies conducted in various countries have shown the mycoplasma infection rate among cultures in different laboratories to vary from 15% to 80% and, in some, to even reach 100% [3, 5].

Mycoplasma is an umbrella term for representatives of the Mollicutes class, the smallest bacteria lacking a cell wall and capable of self-reproduction. The small genome size limits the biosynthetic abilities of these microorganisms and defines their parasitic way of life. The great attention to mycoplasma is nowadays dictated, on the one hand, by the study of the molecular patterns of minimal cellular function sand, and on the other hand, by practical necessity. Mycoplasmas parasitize humans, animals, and plants, where some of them are agents of socially significant diseases, and the main contaminants of cell cultures and vaccines. Control over mycoplasma infection is a serious problem, the solution to which can probably be found in the molecular mechanisms of adaptation that allow mycoplasma to survive under various conditions and to overcome the protection barrier of higher eukaryotes and their persistence [1-3, 6-8].

## MYCOPLASMAS ARE THE MAIN CONTAMINANT OF CELL CULTURES

The significant amount of theoretical and practical data accumulated recently has dramatically changed our no tion of mycoplasma pathogenicity. It has become clear that bacteria have elaborated sophisticated mechanisms to survive under severe conditions and remain virulent [9-18], whereas the conditions of *in vitro *cultivation of eukaryotic cells favor mycoplasma growth [13, 19]. Together with cells from the original organisms, whose tissues are used to create an *in vitro *culture, researchers themselves, as well as components of the medium and laboratory facilities, can act as a source of mycoplasma contamination. In this context, all representatives of Mollicutes are considered to be potential contaminants of cell cultures. At the moment, there are almost as many as 30 types of mycoplasmas that have been identified in cell cultures, whereas 95% of cases are caused by the following 6 mycoplasmas: *Mycoplasma arginini*,* M. fermentans*, *M. hominis*, *M. hyorhinis*, *M. orale *and* Acholeplasma laidlawii *[2, 3]. This knowledge allows one to assume that these bacteria possess special features that define their prevalence in their ecological niche, and, consequently, that contamination can be controlled through the adaptation mechanisms of mycoplasmas.

*A. laidlawii *is a mycoplasma species that appears to have unique adaptation abilities. This widely spread type is the agent of phytomycoplasmosis [1, 20, 21]. Although it is present in humans and animals in various pathological processes, there has been no reliable evidence of its pathogenicity so far [1, 3, 5]. Mapping of the *A. laidlawii *genome carried out in Russia [22] have made it possible to establish the adaptation mechanisms of this mycoplasma using post-genomic technologies. Genomic, transcript, and proteomic profiling, along with the nanoscopic analysis, have allowed researchers to identify the stress-reactive proteins and genes of *A. laidlawii*. It has been demonstrated that the mechanisms of mycoplasma survival under severe conditions, as well as the mechanisms of formation of host– parasite relationships and virulence, are connected to the secretion of extracellular vesicles by this bacteria [16, 20, 21, 23, 24].

**Fig. 1:**
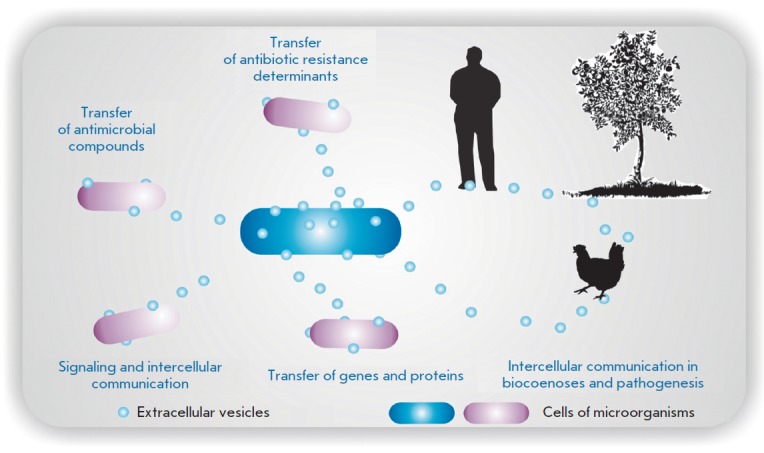
Extracellular vesicles mediate the traffic of a broad range of components, transfer of virulence determinants, and development of resistance to antimicrobial agents; they participate in signaling, intercellular communication, and pathogenesis [41]

Extracellular membrane vesicles mediate the common secretion mechanism in prokaryotes and eukaryotes and constitute an important part of the bacterial secretome [25]. Along with the membrane components, they may contain cytoplasmic proteins, toxins, DNA, and RNA [26, 27]. Discovered in gram-negative bacteria several decades ago, extracellular vesicles were recently been found in archaea [28], gram-positive bacteria [29], and in the smallest wall-less prokaryotes; namely mycoplasma [16, 24]. Vesicles were shown to play an important role in cell-to-cell communication as carriers of essential cell-specific information [25, 30- 32]. The internalization of these nanostructures triggers cell-target reprogramming, which can be detected by proteomic and transcript analyses [33, 34]. Bacteria-secreted extracellular vesicles mediate the protein traffic and transfer of virulence determinants, participate in the formation of the host–parasite system and that of the resistance to antibacterials and, respectively, in the adaptation to different environmental conditions (*Fig. 1*) [25, 27]. In accordance with the virulence criteria, the extracellular vesicles of pathogenic bacteria belong to a new type of infectious agents, which makes it necessary to adjust current approaches to the control of bacterial infections [21, 31, 35].

**Fig. 2:**
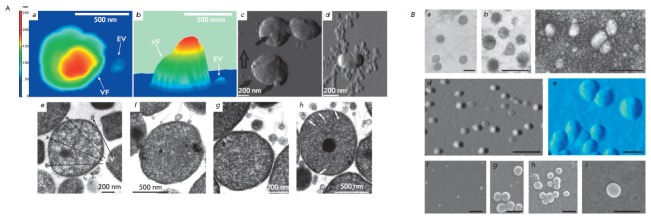
Atomic force (A, B, C) microscopy and transmission electron microscopy images (E–G) of the cells of a A. laidlawii PG8 culture and atomic force microscopy images of M.gallisepticum S6 cells (D) EV – extracellular vesicle; VF – vegetative forms. Transmission electron (A, B, C (negative staining)), atomic force (D, E) and scanning electron microscopy images (E–I) of extracellular vesicles of A. laidlawii PG8. The scale bar is 200 nm

**Fig. 3:**
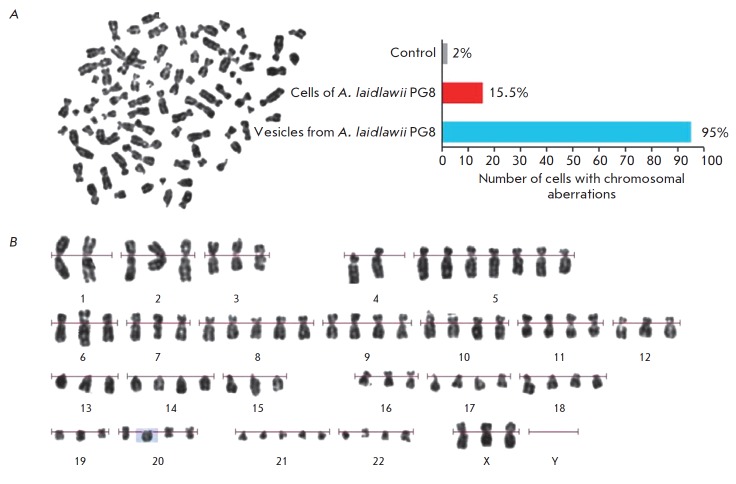
Metaphase plate (A) and karyogram (B) of human peripheral blood lymphocytes after the cells were incubated with vesicles of A. laidlawii PG8

**Fig. 4:**
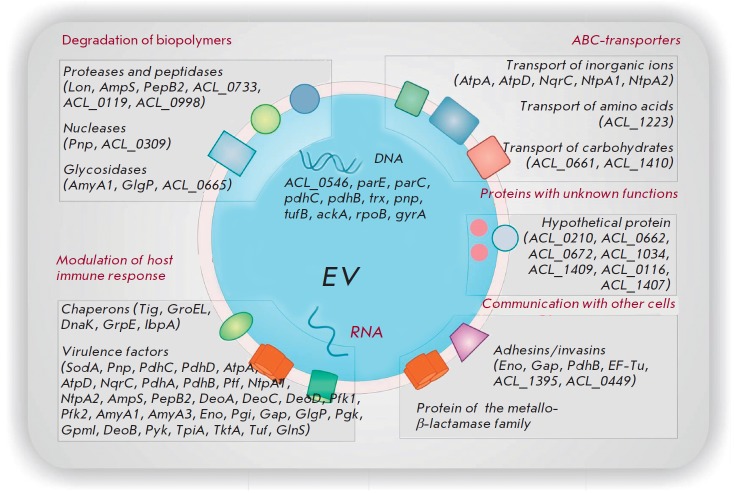
Extracellular vesicles of A. laidlawii PG8 contain a specific set of DNA and RNA nucleotide sequences

*A. laidlawii *cells have been shown to secret vesicles (20–120 in diameter) into the intracellular space under different growth conditions; however, the vesicle generation rate considerably increases under stress (*Fig. 2*). Vesicles determine such virulent properties of mycoplasma as infectivity, invasiveness, and toxigenicity; they also induce the clastogenic effect in eukaryotic cells *in vitro* (*Fig. 3*). Vesicle penetration precedes mycoplasma invasion of plant tissues, destroys their ultrastructure, induces modulation of gene expression and protein synthesis in infected organisms, and mediates the development of mycoplasma resistance to antibacterials [16, 20, 21, 24, 36]. Global proteomic profiling has allowed researchers to “make an inventory” of the proteins of *A. laidlawii *extracellular vesicles PG8 secreted in an axenic culture [37]. It turns out that most polypeptides exported from mycoplasma cells with vesicles are virulence factors including adhesins, enzymes of a protein, polysaccharide, and nucleic acid degradation (*Fig. 4*).

In addition to membrane components and cytoplasmic proteins, the extracellular vesicles of *A. laidlawii* PG8 contain a specific set of nucleotide sequences that can be used as markers of bacterial vesicles in analyzed species [20, 24, 36]. Similar data on the structure and composition of extracellular vesicles were obtained for* M. gallisepticum* (*Fig. 2*), a widespread agent of avian diseases and the main contaminant of viral chick embryo vaccines [24]. The results indicate that vesicular traffic associated with extracellular membrane vesicles in archaea, classic gram-positive, and gram-negative bacteria was also found in the smallest wall-less prokaryotes. This fact makes it necessary to reconsider our understanding of the interaction between the mycoplasma and the cells of higher organisms and to design a strategy for controlling infectious agents.

## MYCOPLASMA CONTAMINATION CONTROL

Since mycoplasmas do not have a rigid cell wall, close contact between the cytoplasmic membrane of the host and that of the parasite is possible; under certain conditions, this may cause cell fusion [1, 38]. Some mycoplasmas have specific organelles at their poles (the so-called tips or blebs) that mediate gliding motility and adhesion between bacteria and the eukaryotic cell membrane [1, 39]. Adhesion can be accompanied by invasion of the cell [3]. However, even when staying on the surface and thus in close contact with the host cell membrane, mycoplasmas induce modulation of the genome expression and cause considerable changes in the metabolism in eukaryotic cells [3, 38]. A series of studies aimed at determining the patterns of transcription profile modulation in cell cultures upon mycoplasma contamination show that the latter triggers changes in the expression of a broad range of genes in the host cell (*Table 1*). The genes whose expression changes include a significant portion of the most important ones encoding regulatory proteins, such as oncogenes, tumor suppressor genes [40], cytokines [41], receptors, and components of signaling pathways [42]. Changes in the expression may become overt as soon as several hours after inoculation [42], whereas prolonged cultivation of inoculated cells (18 weeks) may lead to their irreversible transformation to the extent of malignant degeneration [40]. The nature of transcript profile modulation in inoculated cells varies substantially depending on the mycoplasma type, cell culture type, multiplicity of infection, and cultivation period. Thus, contamination with mycoplasmas makes it impossible to adequately evaluate the results obtained using an inoculated culture. In particular, the effect of compounds suggested as promising pharmaceutical agents cannot be studied.

**Table 1:** Change of mRNA expression of a number of genes in cells inoculated with mycoplasma in 3-7 days after contamination

Mycoplasma	Cell culture	Induction of mRNA expression	Suppression of mRNA expression	Reference
M. fermentans	Epithelial cells of prostate HPV E7	14 cytokines	TGFβ1, TGFβ3	[41]
M. genitalium		12 cytokines	GM-CSF, IL-1Ra, M-CSF	
M. hominis		12 cytokines	TGFβ2	
M. penetrans		14 cytokines	TGFβ2	
M. fermentans	Epithelial cells of cervical canal HPV E6	17 cytokines	0	[41]
M. genitalium		13 cytokines	G-CSF, IL-1Ra	
M. hominis		13 cytokines	IL-1α, IL–1β	
M. penetrans		15 cytokines	TGFβ2,TGF-β3	
M. synoviae	Chicken macrophages MDM	Cytokines, lysozyme, apoptosis inhibitor, 11 enzymes, 4 types of receptors, 10 proteins of the signaling system	ovotransferrin, glutathione S-transferase, guanylate-binding protein	[42]
M. fermentans incognitas	Mice embryoblast C3H	92 genes encoding oncogenes and tumor suppressors	43 genes encoding oncogenes and tumor suppressors	[40]
Phytoplasma	Paulownia culture	769 genes	437 genes	[45]

Despite the fact the hundreds of genes whose expression changes upon contamination of eukaryotic cells with mycoplasma have been identified [41-45], no common markers of mycoplasma contamination have been found. Mycoplasmas may trigger the activation of macrophages cultivated i*n vitro*, suppression of antigen presentation, modification of the immune reactivity, signal transduction, viral proliferation, and apoptosis [40, 46-54]. Mycoplasma contamination may remain unnoticed for a rather long time; visible changes appear only at high multiplicity of the infection [1, 3]. The most serious effect of contamination is the loss of the cell culture due to the growth of microorganisms and, respectively, the irreversible worsening of the condition of the cells. Depending on the mycoplasma species, cell line and cultivation conditions, one may observe various cytopathic reactions, including, for instance, chromatin condensation, leopard cells, chromosome aberrations, suppression of cell division, and deprivation of cell culture growth [3, 5]. The main reason for these reactions is mycoplasma interference with cell metabolism, competitive absorption of nutrients and release of bacterial toxins, enzymes of protein, and DNA and RNA degradation [1, 38]. The extracellular vesicles of mycoplasma may actively participate in these processes. We have demonstrated in a series of special experiments that the RNA activity of *A. laidlawii *PG8 and that of *M. hominis *PQ37 account for 86% and 89%, respectively, of the overall activity of the cellular and extracellular RNases of these bacteria [55]. The ribonucleic activity of the secreted vesicles may to a large extent determine the genotoxic properties of these contaminants revealed earlier [56-58]. Taking into account the cytotoxic potential of numerous bacterial RNases [59-61], one may assume that the cytopathic reactions of contaminated cell cultures are substantially determined by the activity of the vesicular RNases of their mycoplasmas. The revealed high RNase activity of mycoplasma vesicles determines the apoptotic effect of these enzymes on the target cells of the mycoplasma vesicular traffic.

Since mycoplasmas may influence almost all the parameters of eukaryotic cells, the results obtained with infected cells should be treated with suspicion. Due to this fact, the editors of journals suggest that authors provide results of the verification of the experimental data (in particular, cell lines) for mycoplasma contamination. Since many viral vaccines are created using a primary cell culture, the problem of their contamination with mycoplasma is of special importance as vaccine contamination poses a potential risk to human health [1, 3, 5]. In this regard, many countries demand that products created using primary cell cultures, such as viral vaccines against measles, rubella, poliomyelitis, rabies, mumps and some others, be thoroughly checked for mycoplasma contamination [3].

Thus, mycoplasma contamination of cell cultures is a serious problem both for fundamental studies and applied research. It is clear that all cell lines being purchased should undergo strict control for mycoplasma contamination before they reach a laboratory, whereas the cultures that are already in use should be regularly checked. The discovery of extracellular vesicle traffic in mycoplasma makes it necessary to control new-type infectious agents as well.

**Methods for mycoplasma detection**

There are no common markers of cell contamination with mycoplasma. Among specific diagnostic tools (*Table 2*), there are three approaches recommended by international expert organizations.

**Table 2:** Methods used to detect mycoplasma in cell cultures

Microbiological cultivation^*^
Electronic microscopy
Biochemical assays
Detection of adenosine phosphorylase activity (6-MPDR)
Enzymatic conversion АТР → АDP detected by luciferase
Chromatographic detection of the transformation of radioactively labeled uridine to uracil with the uridine phosphorylase of mycoplasma
Immunoassays
Immunofluorescence
ELISA
Molecular biology tests
Hybridization analysis
Dot-blot hybridization with specific probes
PCR, RT-PCR ^*^
Microscopic detection
Direct staining of DNA with fluorescent dye (DAPI, Hoechst 33258) ^*^
Fluorescent in situ hybridization (FISH) using probes labeled with fluorescent dyes

^*^ – officially approved by a number of international expert organizations: FDA Points to Consider (May 1993), Regularien 21CFR610.30; USDA federal code #9CFR113.28; United States Pharmacopoeia, (USP 33/NF 28 '63' and '1226', Mycoplasma tests, 2010); European Pharmacopoeia (EP 2.6.7., Mycoplasmas, 7th ed.; 2012); Japanese Pharmacopoeia (JP); ICH Guideline for biotechnological/biological products.

Microbiological cultivation is the main approach to detect mycoplasma [3, 62]. In this analysis, an aliquot of the cell culture supernatant is added to a liquid medium to cultivate mycoplasmas. After several days of incubation, the culture is transferred to an agar plate containing the same components as the medium. The plates are then incubated for some time (up to 2 weeks) under aerobic conditions at 37 °C. The emergence of two-phase “fried-egg” colonies indicates that mycoplasmas are present in the test samples. This test is theoretically highly sensitive, but it requires a lot of time (up to 4 weeks) and expensive media. Furthermore, many types of mycoplasmas poorly grow on cell-free media, whereas some of them are impossible to grow* in vitro *[1, 62]. In this test, the medium can also become infected from the outside: either from a researcher, medium components, or laboratory facilities. Thus, this detection method includes the risk of obtaining false-positive and false-negative results. Moreover, the cultivation procedure does not allow one to reveal the extracellular vesicles of bacteria.

The second recommended approach to detect mycoplasma contamination is staining DNA with fluorescent DAPI or Hoechst 33258 [3, 62, 63]. This test is very simple and does not require much time; the result can be obtained in as early as 2–3 hours. However, certain parameters of the condition of cell culture may lead researchers to a wrong decision about whether the culture is contaminated with mycoplasma or not. For instance, extracellular vesicles secreted by eukaryotic cells in a mycoplasma-free culture contain DNA and RNA, which significantly complicates the interpretation of the results, whereas administration of antibiotics makes it impossible to use the proper test. Nevertheless, this approach is very popular due to its simplicity and the possibility to use it for detecting uncultivable mycoplasmas or those growing poorly on cell-free media. In this analysis, the test culture supernatant is added to a mycoplasma-free indicator cell culture (lines Vero B4, NIH 3T3 or 3T6) [64]. Cells are grown in flasks containing sterile slips, which are washed and stained with fluorescents after several days of cell culture growth. In this case, prolonged duration of the test poses a risk that contaminants would spread in the laboratory.

Polymerase chain reaction (PCR) is nowadays the most effective way to detect mycoplasma [1, 3, 62, 65, 66]. PCR variants allow one to detect mycoplasma DNA and RNA. Oligonucleotides for the amplification of variable regions of 16S rDNA or rRNA and sequences of 16–23S intragenic regions are usually used as primers. PCR can include either a single amplification cycle or the nested PCR with two pairs of primers. The latter variant increases test sensitivity and specificity, but at the same time it poses a risk of obtaining false results due to the possible contamination of target DNA. In addition, the medium components can be Taq polymerase inhibitors: so the test should be carried out using extracted DNA rather than the raw lysate of the cell culture supernatant. Administration of antibiotics may lead to false results: so the culture should be grown without antibiotics for at least 2 weeks before performing the test.

The use of reverse transcription PCR (RT-PCR) to detect rRNA increases test sensitivity; however, this variant is labor-intensive. Taking into account the fact that mycoplasma titer in cell cultures is sufficient to register bacterial DNA, a simple one-step PCR is acceptable. It meets the requirements for a short-term test: it is easy to perform, highly sensitive, specific, and cost-effective. Meanwhile, positive PCR results do not necessarily mean that the sample has the living cells of a contaminant (which is important to keep in mind while analyzing the material after the measures aimed at mycoplasma eradication). In certain cases, when PCR testing of the culture analyzed for mycoplasma contamination yields positive results, amplicon sequencing is needed to draw the final conclusions. Nevertheless, PCR has been approved by international expert organizations, and nowadays there are enough commercial sets for testing cultures for mycoplasma contamination available on the market [3, 62]. The primers used in these sets are ineffective in detecting extracellular vesicles; however, the discovery of mycoplasma-specific nucleic acid sequences in vesicles [20, 21, 24, 36] presents a challenge for developing PCR tests that would detect the corresponding infectious agents.

In addition to the officially approved approaches that have been listed above, there also are other methods: immunoassays and hybridization tests that in addition to using antisera, monoclonal antibodies, and DNA–RNA hybridization employ radioactive or fluorescent tags; biochemical and microscopic methods, etc. (*Table 2*) [1, 3, 43, 62, 67, 68]. All these methods are characterized by different sensitivities and are not free of the disadvantages typical of the aforementioned approaches.

The data presented above is evidence that the problem of detecting mycoplasma contamination has yet to be solved. All the available methods have disadvantages and limitations, so it is recommended that a cell culture be simultaneously tested using several techniques [1, 3, 62]. It is clear that in order to test the medium components for the presence of such infectious agents as extracellular bacterial vesicles, special tests against the markers of these organelles need to be elaborated. Detection of common marker sequences to reveal the respective infectious agents implies a complex study of extracellular vesicles in various Mollicutes species. Only the first steps have been made in this direction so far [16, 20, 36, 37].

## METHODS FOR MYCOPLASMA ERADICATION

Elimination of the infected cell culture and obtainment of a new, clear one is believed to be the best way to solve the problem of mycoplasma contamination [1, 3, 69]. If this is impossible, then one is faced with the decontamination issue, which means mycoplasma eradication without damaging eukaryotic cells. However, despite the fact that numerous approaches for the elimination of mycoplasma have been suggested and tried over several decades, an effective one has not been found yet. Nevertheless, researchers have remained persistent, and successful cases of cell culture decontamination by virtue of either new or modified approaches are reported from time to time [1, 3, 69-71]. The most popular one is the use of antibiotics.

Specific features of mycoplasma biology define the pattern of their susceptibility to antibiotics. Many of those turn out to be inefficient as mycoplasmas lack targets they are aimed at. For instance, they lack cell wall peptidoglycan whose synthesis is inhibited by penicillin [1, 3, 72]. On the other hand, some antibiotics do not cause mycoplasma death, but they slow down its growth and thus disguise the presence of a contaminant [2]. This fact is the reason why antibiotics are not recommended for prophylactic use upon *in vitro *cultivation [2, 5, 69]. Nonetheless, researchers continue to look for agents for cell culture decontamination among antibiotics [2, 3, 67, 69].

Three groups of antibiotics exhibiting some activity against mycoplasma are known thus far: macrolides, quinolones, and tetracyclines [3, 69, 72]. It has been reported in a number of publications that serial treatment of cell cultures with certain combinations of antibacterial agents belonging to these groups effectively removes mycoplasmas [3, 67, 69]. However, experimental attempts to decontaminate cell cultures according to the reported protocols often fail [1, 71, 73]. Taking into account this fact, together with the negative impact of antibiotics on cell cultures, most researchers remain skeptical of attempts to eradicate mycoplasma with antibiotics, while commercial companies continue to actively advertise these products.

A significant problem of antibiotic therapy against mycoplasma infections is that mycoplasmas quickly develop resistance [1, 19, 74]. The mechanisms of rapid development of resistance to antibiotics are not clear. It is assumed that, alongside the known mechanisms of developing resistance to such antibiotics as quinolones, the mycoplasmas use other mechanisms that have not been identified yet [75-77]. Extracellular vesicles have recently been reported to potentially mediate the mechanisms of developing resistance to antibiotics in bacteria [78, 79], including mycoplasma [36]. Involvement of extracellular vesicles in the formation of mycoplasma resistance to antibiotics has been proved for* A. laidlawii. *To prove it, we used mycoplasma strains characterized by different susceptibilities to ciprofloxacin: the laboratory (PG8) and PG8R, which was derived from it in a stepwise manner and showed high resistance to the antibiotic. It turned out that these strains also had different clearance mechanisms and different vesicle generation rates. It was found that the high resistance of a PG8R strain is associated with a high vesicle generation rate and that vesicles, in turn, participate in the ciprofloxacin traffic exhibiting a bacteriostatic effect towards *Staphylococcus aureus* (a strain sensitive to the antibiotic)*. *The strain with high resistance to ciprofloxacin was found to have a C R T transition at the 272 position (causing a serin to leucin transition –Ser (91) Leu in the target protein molecule) in *parC *locus (determining resistance to fluoroquinolone) of the target gene (topoisomerase IV). It turned out that the vesicles of this mycoplasma strain export the mutant gene of the target protein. Export of the antibiotic target genes mediated by extracellular vesicles favors a quick distribution of the mutant target of quinolones over the microbiocenosis by horizontal transfer [80]. Performance of this pattern has been recently demonstrated in model systems of *Escherichia coli *and *Pseudomonas aerogenosa *[81, 82]. The study of these processes in mycoplasma has not been completed yet, although it is already clear that extracellular vesicles are the important component of the mechanisms of quick adaptation to antibacterial products. Considering the fact that vesicle secretion is the process that allows microorganisms to survive under various conditions [27, 32], searching for effective antibiotic means of cell culture decontamination does not appear promising.

Thus, mycoplasma contamination of cell cultures and mycoplasma diagnosis and elimination remain serious problems [1, 3, 7, 69, 83, 84]. It is absolutely clear that reliable methods for detecting infectious agents and decontamination methods are needed, which would be based first and foremost on a thorough investigation of mycoplasma genetics and physiology. The discovery of the extracellular vesicular traffic in mycoplasmas mediating cell-to-cell interactions and pathogenesis makes it necessary to take into account new infectious agents. Since cell cultures are used to produce vaccines and physiologically active compounds, quickly solving the discussed issue is topical both for fundamental science and the biotechnological production of pure, next-generation products.
